# Examining the Naringin Content and Sensory Characteristics of Functional Chocolate Fortified with Grapefruit Peel Extract

**DOI:** 10.1007/s11130-023-01091-5

**Published:** 2023-08-18

**Authors:** Zsolt Ajtony, Beatrix Sik, Aron Csuti

**Affiliations:** Department of Food Science, Albert Casimir Faculty at Mosonmagyaróvár, Széchenyi István University, 15-17 Lucsony Street, Mosonmagyaróvár, 9200 Hungary

**Keywords:** Functional food, Grapefruit peel, Naringin, Chocolate, HPLC

## Abstract

Grapefruit peel contains a high concentration of naringin- a potent antioxidant with strong bioactive properties. In this study, a new type of functional chocolate fortified with grapefruit peel extract and different concentrations of aqueous methanol and ethanol were evaluated as extraction solvents. A new high-performance liquid chromatography (HPLC) method to analyze the naringin content of the fortified chocolates was developed with a recovery of 107% ± 3.1% and repeatability below 3.5%. A sensory evaluation was conducted to assess the preference for the chocolates among individuals who self-described a preference for bitter flavors. No significant preference was observed in the cases of astringency and aftertaste while the increased bitterness proved to be favorable. However, taste, flavor and overall acceptability were regarded somewhat less favorably. While chocolate proved to be a satisfactory carrier for naringin and had several enjoyable characteristics, further research may focus on improving the organoleptic properties of chocolates fortified by naringin.

**Supplementary Information:**

The online version contains supplementary material available at 10.1007/s11130-023-01091-5.

## Introduction

Naringin is a polyphenol and potent antioxidant abundant within the peels of grapefruit (*Citrus paradisi*), pomelo (*Citrus grandis*), and sour orange (*Citrus aurantium*). The molecule is a flavanone-7-O-glycoside between a naringenin aglycone and a neohesperidose moiety [1]. This compound has been shown to protect against neurodegenerative conditions such as Alzheimer’s disease [2] cardiovascular diseases, and diabetes [3] while also exhibiting hepatoprotective, anti-obesity [4] nephroprotective [5, 6] anti-inflammatory [7]and anti-carcinogenic characteristics [8, 9]. Naringin is generally recognized as safe [1] and no significant toxic effects have been found [9]. Additionally, the NOAEL of naringin was found to be > 500 mg/kg of body weight when tested on Beagle dogs [10]. However, it is important to note that ingesting naringin- after being hydrolyzed into naringenin- can cause adverse drug interactions. Thus, the FDA has begun to put warning labels on certain medications calling their users to avoid grapefruit products [11].

Research into foods fortified with polyphenols is rapidly increasing due to their availability and prophylactic activity. Given the aforementioned bioactive properties, grapefruit peel and naringin have been used as an ingredient in a several types of foodstuff such as cakes [12, 13], cookies [14, 15] mustard oil [16], margarine beverages [17]. Additionally, several studies have evaluated the potential of citrus peel as a functional food ingredient [18, 19]. However, to the best of our knowledge, grapefruit peel has not been utilized as a functional ingredient in chocolate. Dark chocolate may well serve as an excellent carrier for plant extracts because it can easily mask its taste and color while allowing for even distribution within the food structure. Additionally dark chocolate has a rich phenolic profile by itself, containing large quantities of flavan-3-ols such as catechin and epicatechin. As with naringin, these compounds also have anti-inflammatory properties and attenuate risk factors for cardiovascular disease [20, 21]. For this reason, the goal of this study was to produce chocolate fortified with grapefruit peel extract and to develop a novel HPLC method for analyzing the naringin content of the product.

## Materials and Methods

The materials and methods used in this work are reported in detail in the Supplemental Material section.

## Results and Discussion

### HPLC-Method Validation

To validate our method, we evaluated the resolution, number of theoretical plates, retention time, and peak asymmetry of the method by injecting three replicate standards and chocolate extracts. The determination coefficient (R^2^) of the calibration curve of naringin was 0.9999, which indicates the linearity of the HPLC method. The LOQ was found to be 4 µg/mL, while the LOD was estimated to be 1.2 µg/mL. Recovery was determined to be 107% ± 3.1% while the repeatability and reproducibility of our method was evaluated by examining the relative standard deviation (RSD%) of naringin within the chocolate. The repeatabilities (intra-day precisions) determined by triplicate measurements on four various days were 3.7, 0.4, 3.5, and 0.7%. The reproducibility (inter-day precision) calculated from 4 triplicate measurements (twelve replicates) was found to be 3.5%. Sensitivity was recorded as 6.586 mL/µg. Therefore, these results indicate that the used method was adequate for the quantitative and qualitative determination of naringin in chocolate.

### Effect of the Solvent Composition on the Extraction Yield

**Fig. 1:**
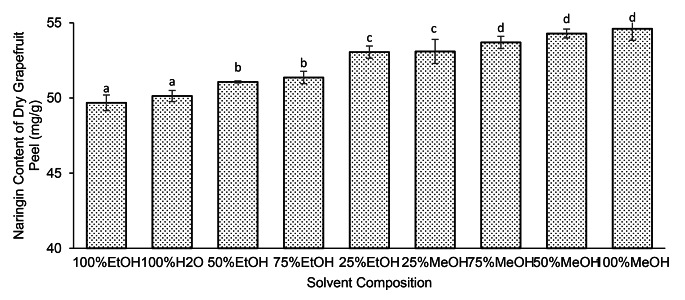
Effect of solvent composition on naringin yield from grapefruit

To determine the most effective solvent for the extraction of naringin from grapefruit peel, we made triplicate measurements of wet grapefruit peel extracts prepared immediately before injection. While doing so, the solvent composition was varied. As seen in Fig. 1, solvent compositions of 25, 50, 75, and 100% aqueous ethanol and methanol were tested. A visualization of our results can be seen in Fig. 1.

Confidence intervals refer to standard deviation. Lettering shows which solvents produced statistically identical outcomes.

As shown in Fig. 1, pure methanol was proven to be the best extraction solvent while pure ethanol and deionized water yielded the lowest concentration of naringin. However, methanol is well-known to be highly toxic and the aqueous solvents were difficult to remove from the extract. Therefore, ethanol was chosen as an extraction solvent for the production of chocolates used for sensory evaluation. The observed superior solvency of methanol was in line with the findings of Feng et al. [22] where the extraction efficiency of methanol and ethanol for naringin from oranges were compared. However, ethanol is still commonly used due to its nature as a food-grade solvent and overall effectiveness [23].

### Characterization of Grapefruit peel Extract

The grapefruit peel had a dry matter content of 22.3% which was determined gravimetrically. The grapefruit peel extract was a dull, red-orange powder comprising 61.9%, 57.9%, and 42.95% of dry peel mass when extracted with methanol, ethanol, and 75% ethanol respectively. The measured naringin content of the dry extract was 130 ± 2.3 mg/g when using ethanol, 111.9 ± 1.6 mg/g when using methanol, and 96.2 ± 1.4 mg/g when using 75% ethanol. The extraction yield from the dry peel was 54.5 mg/g with methanol, 45.6 mg/g with ethanol, and 53.6 mg/g with 75% ethanol. This was in line with the findings of Li et al. [24] (52.03 mg/g when using methanol) showing that our extraction method provided sufficient yield. As highlighted by Sharma et al. [9], and Li et al. [25], naringin poses no health risks below 2 g per person or in a dose of less than 200 mg/kg in body weight.

### HPLC Analysis of Fortified Chocolate

**Fig. 2:**
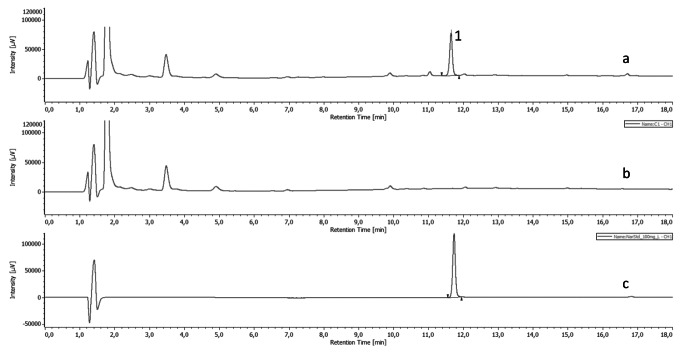
HPLC analysis of fortified and control chocolates. Chromatogram depicting naringin peak (1) in fortified chocolate (a) control chocolate (b) and 100 ?g/mL naringin standard solution (c). Naringin is absent from control chocolate

The protocol for chocolate production and analysis was based on the method used by Sik et al. [26]. Naringin and naringenin were both detected at 280 nm during the procedure. Our experiments showed that pure methanolic extraction resulted in the highest naringin yield (54.5 mg/g) from grapefruit peel so methanol was chosen for the extraction of naringin from the fortified chocolate. Methanolic solvents are commonly used to extract polyphenols from chocolate matrices as seen in studies by Belščak-Cvitanović et al. [27], Poliński et al. [28], and Carvalho et al. [29]. Fig. 2 shows the chromatogram measured for chocolates and the standard solution.

As seen from the chromatogram, naringin was successfully separated from the other minor components of the fortified chocolates. In addition, the measured concentration was 1.04 mg/g with 101 ± 3.4% recovery. This data highlights that dark chocolate provides a suitable matrix for fortification with naringin. Other authors have also examined the potential for chocolates to be fortified with fruit extracts offering proof of the suitability of chocolate to serve as a medium for functional ingredients. For example, Kaur et al. [30] produced a meat chocolate fortified with blueberry and raspberry extract and observed improved microbiological quality and boosted antioxidant activity. Polinski et al. [31] also measured an increase in phenolic content and antioxidant capacities when making dark chocolates fortified with chokeberry, elder flower, and elderberry extracts.

### Effect of Extraction time on Naringin Yield

**Fig. 3:**
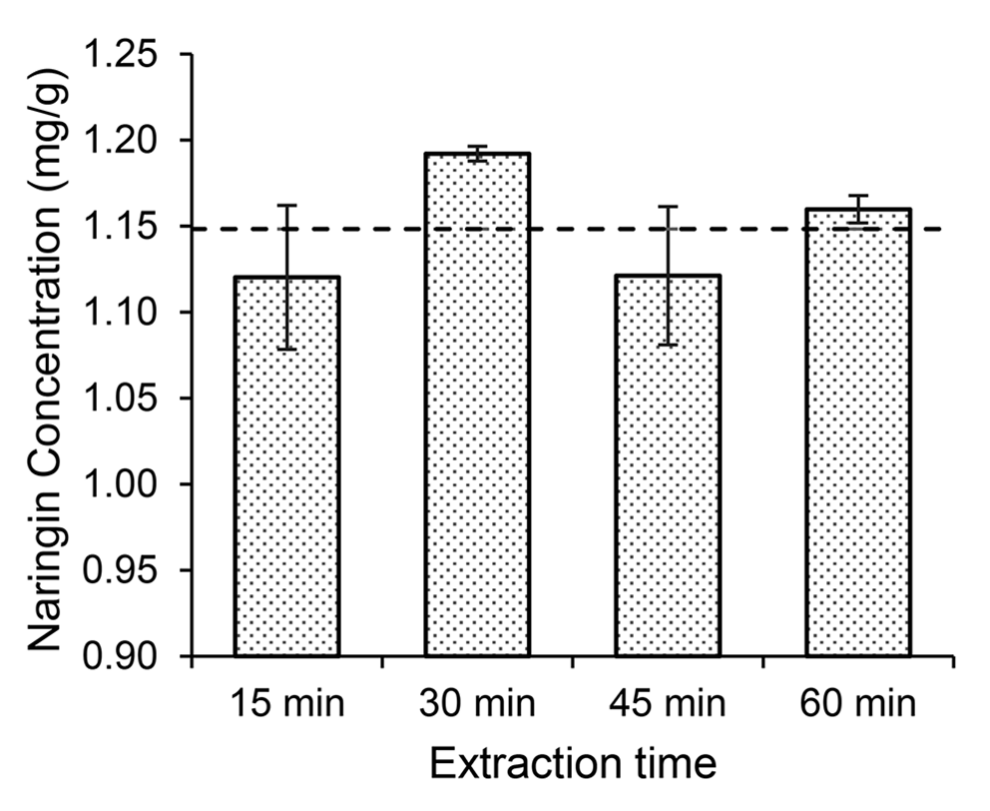
Effect of extraction time on naringin yield. Horizontal Dashed line Represents Average Concentration, Confidence Intervals Represent Standard Deviation (SD)

The amount of naringin that could be extracted from the chocolates as a function of time was also investigated. We also investigated the factor of extraction time during the extraction of naringin from chocolate. As seen in Fig. 3, extractions of 15, 30, 45, and 60 min provided a statistically identical result.

### Results of Organoleptic Evaluation

**Fig. 4:**
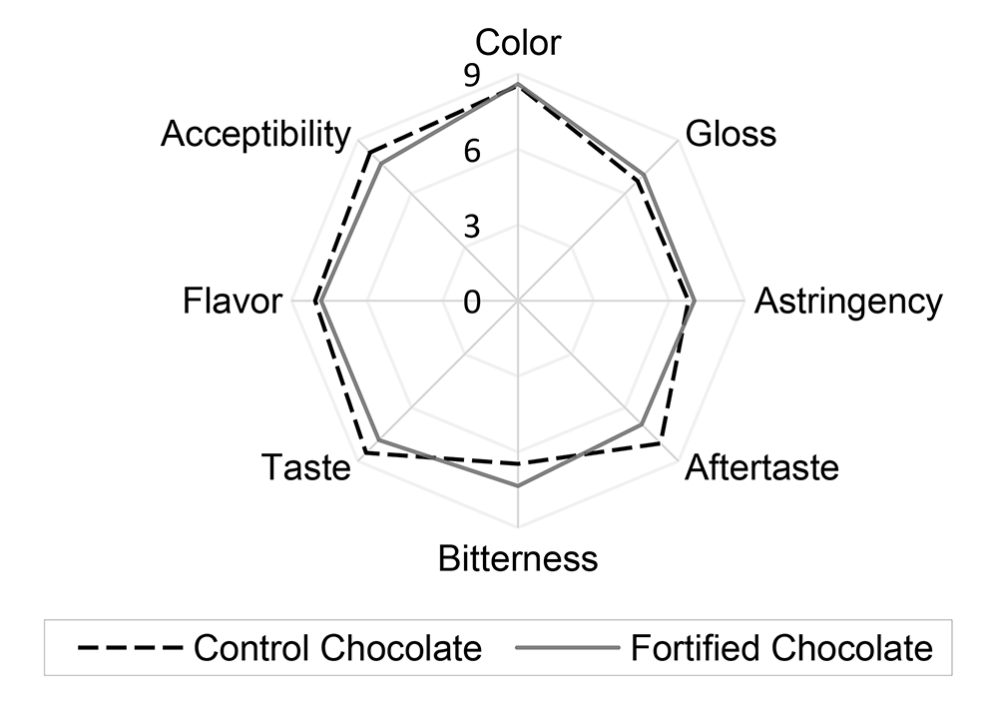
The sensory evaluation of fortified and non-fortified chocolates 0 represents the most negative appeal of a characteristic while 9 represents the strongest appeal of a characteristic

Due to the strong change in the organoleptic properties caused by the fortification with grapefruit peel extract, the sensory evaluation was focused towards individuals who enjoy bitter flavors. However, the evaluators showed no significant preference for either chocolate in the categories of aftertaste, or astringency. In fact, a clear preference was shown for the enhanced bitterness of the chocolate. However, taste, flavor and overall acceptability was greater in the case of the control chocolates. A summary of the organoleptic trials can be seen in Fig. 4.

There was no significant difference found within the evaluation of the visible traits of the chocolate. Even though the increase in bitterness may be welcome for some, further research may focus on improving the sensory characteristics of fortified chocolates. A possible solution to this issue may be using encapsulation technologies, which may decrease the changes in flavor while maintaining or increasing the antioxidant activity and bioavailability of naringin [32, 33]. Alternatively, naringin may be hydrolyzed to its less-bitter aglycone moiety naringenin through the use of naringinase, or a combination of α-L-rhamnosidase and β-glucosidase [34].

## Conclusion

Our study evaluated chocolate as a potential oral delivery system of naringin. Ethanol was chosen as to extract naringin from grapefruit peel due to its volatility and lesser toxicity when compared to methanol. A simple, rapid, accurate and precise HPLC-DAD was developed to quantify naringin in the fortified chocolate. While increase in bitterness was found to be appealing to some, further studies may seek to minimize organoleptic changes through the addition of sweeteners, nanoencapsulation, or the enzymatic hydrolysis of naringin.

## Electronic Supplementary Material

Below is the link to the electronic supplementary material.

Supplementary Material 1

## Data Availability

The datasets generated during and/or analyzed during the current study are available from the corresponding author on reasonable request.
