# Supplementary material for: Examining the Naringin Content and Sensory Characteristics of Functional Chocolate Fortified with Grapefruit Peel Extract
Source: Plant Foods Hum Nutr. 2023 Aug 18;78(3):533–8. doi: 10.1007/s11130-023-01091-5 (PMC10495487; doi:10.1007/s11130-023-01091-5)
Supplement: Supplementary file 1 — Supplementary Material 1 [file 11130_2023_1091_MOESM1_ESM.docx]

**Supplementary Material – Plant Foods for Human Nutrition**

**Examining the Naringin Content and Sensory Characteristics of Functional Chocolate Fortified with Grapefruit Peel Extract**

**Zsolt Ajtony, Beatrix Sik*, Aron Csuti**

Department of Food Science, Albert Casimir Faculty at Mosonmagyaróvár, Széchenyi István University, 15-17 Lucsony Street, 9200 Mosonmagyaróvár, Hungary

***Corresponding author: Beatrix Sik (**[sik.beatrix@sze.hu](mailto:sik.beatrix@sze.hu)**)**

**ORCID**

Beatrix Sik <https://orcid.org/0000-0002-1786-2710>

Zsolt Ajtony https://orcid.org/0000-0002-6484-5147

Aron Csuti https://orcid.org/0000-0002-5681-6055

Materials and reagents

The organic grapefruits (Citrus paradisi) were bought at local stores during the summertime of 2021. The purchased dark chocolate drops contained 55% cocoa solids and were manufactured on 05.29.2021 with a shelf life of 1 year. The chocolate was composed of sugar, soy lecithin, cocoa butter, fat-free cocoa mass, and natural vanilla. Naringin (Sigma Aldrich, Budapest) with a purity of 95% alongside HPLC-grade methanol, and analytical grade 100% ethanol, 85% phosphoric acid, and n-hexane (Merck, Hungary) were used for the experiments. HPLC-grade acetonitrile (Fischer Scientific, UK) was bought from Reanal (Hungary).

Naringin extract preparation

Prior to extraction, the grapefruits were washed thoroughly and the peels removed and comminuted in a food processor, after which they were freeze-dried and grinded to a powder using a mortar and pestle. Then 40g of freeze-dried and powdered grapefruit peel was refluxed for 30 minutes at the boiling point of 400 mL of extraction solvent (64.7°C, 78.3°C, and 79.5°C for methanol, ethanol, and 75% ethanol respectively). To determine the best extraction solvent composition concentrations of 25%, 50%, 75%, and 100% aqueous ethanol and methanol were tested. The three solvents chosen to prepare extracts for chocolate fortification were the following: pure ethanol, 75% aqueous ethanol, and pure methanol. After cooling, the extraction solvent was removed using a rotary evaporator at 40°C. The dried extract was crushed into powder and stored in an opaque, closed, plastic container under -18°C until it was added to the chocolate.

Fortifying the chocolate

100 g chocolate drops were gently melted in a small glass container over a hot water bath and poured onto a granite slab, where it was mixed with 1.04 and 0.80 g of dry methanolic and ethanolic extracts respectively. We also made chocolate containing standard naringin to calculate recovery. In this case, 88.7 g of chocolate was fortified with 0.10 g of pure naringin. The fortified chocolate samples were placed into a closed plastic container and kept in a freezer. Non-fortified chocolate was used as a control sample for every experiment. All measurements were triplicated throughout our study.

Extracting the naringin from chocolate

The fortified and non-fortified chocolate samples were pulverized in a porcelain mortar and pestle and mixed well. To defat the fortified chocolate, an aliquot of 0.5 g of pulverized chocolate was put into a 50 mL falcon tube and vortexed it with 5 mL of n‑hexane for 2 minutes. The samples were centrifuged for 10 minutes with 3,420 g RCF and the supernatant containing the lipids was removed. The residual n-hexane was evaporated at 30 °C for 60 min in a drying oven. Then the defatted chocolate was vortexed with 30 mL methanol for 2 minutes. Afterward, the extract was placed into an ultrasonic bath at 25°C for five minutes and the volume of the extract was diluted with methanol to 50 mL after being put into a 100 mL glass Erlenmeyer flask and closed with a glass stopper. The flask was then shaken for one hour in a laboratory shaker and the mixture were centrifuged for 10 minutes at 3,420 RCF. The supernatant was filtered through a 0.22 μm PVDF membrane filter and then injected into the HPLC system.

HPLC-DAD analysis

A Gulliver (Jasco , Japan) HPLC system composed of the following units was used for naringin analysis: a PU-980 pump, AS2055Plus autosampler, 7955 column thermostat, LG-980-02 ternary gradient solvent mixer, DG-2080-54 degasser, and a MD-4010 diode-array detector. An Ascentis (Sigma Aldrich, Hungary) C18, 150 × 3 mm, 5µm analytical column at 35°C was used with the following gradient mode: the mobile phase was composed of acetonitrile (A) and 0.05% v/v aqueous phosphoric acid solution (B); 10% to 30% A from 0-15 min, 30% to 50% A from 15-16 min, 50% A from 16-21 min, 50% to 10% A from 21 to 22 min. The flow rate was set to 0.5 mL/min and the injection volume was 2 µL. Data processing was performed by using ChromNav 2.0 Data System (Jasco, Japan).

Validation of analytical method

Our method was evaluated in terms of the limit of detection (LOD), the limit of quantification (LOQ), linearity, precision, and accuracy. To validate linearity, a series of dilutions (from 4 to 250 µg/mL) with standard stock solutions were measured to form a calibration curve. Sensitivity was determined as the slope of the curve. The precision of this method was determined by analyzing each sample three times. Accuracy was assessed by measuring the naringin recovery percentage in chocolate to which a known concentration of naringin standard was added (0.1012 g to 88.74 g chocolate). Percent recovery (%) was then calculated from the results of the analysis. LOD and LOQ were calculated through the following equations: LOD = *3×SD_background_/a*; LOQ = *10×SD_background_/a* respectively. Where *SD_background_* is the standard deviation of the background signal, and *a* is the sensitivity.

Sensory evaluation

The organoleptic evaluation of the fortified and control chocolates was done at the Department of Food Science of Széchenyi István University. The testers included 24 individuals from the ages of 19 to 55. Given the strongly bitter and astringent flavor of the chocolate, subjects who did not have a taste for bitter flavors were screened out. The participants were asked to rate key aspects of the chocolate (color, gloss, astringency, aftertaste, bitterness, taste, flavor, overall acceptability) on a 9-point hedonic scale with 1 signifying strong disapproval and 9 referring to strong approval of the characteristic. Chocolate pieces were served on a white plate and distinguished through alphabetical markers.

Statistical analysis

Experimental results were expressed as the mean of triplicate measurements ± standard deviation (SD). To compare the significance of variations in the data, the ANOVA method was used (P ≤ 0.05). Statistical analyses were performed using LibreOffice Calc 7.0 (The Document Foundation, Berlin, Germany; <https://www.libreoffice.org/>). The significance among the variation of the results recorded within the sensory evaluation were evaluated using a two-tailed, paired Student’s *t*-Test.
